# Global intravascular and local hyperoxia contrast phase-based blood oxygenation measurements

**DOI:** 10.1016/j.neuroimage.2014.07.050

**Published:** 2014-11-01

**Authors:** Ian D. Driver, Samuel J. Wharton, Paula L. Croal, Richard Bowtell, Susan T. Francis, Penny A. Gowland

**Affiliations:** a Sir Peter Mansfield Magnetic Resonance Centre, School of Physics and Astronomy, University of Nottingham, Nottingham, UK; b Cardiff University Brain Research Imaging Centre (CUBRIC), School of Psychology, Cardiff University, Cardiff, UK

**Keywords:** MRI, Oxygen extraction fraction (OEF), Oxygen saturation, Phase imaging, MR susceptometry, Hyperoxia

## Abstract

The measurement of venous cerebral blood oxygenation (Y_v_) has potential applications in the study of patient groups where oxygen extraction and/or metabolism are compromised. It is also useful for fMRI studies to assess the stimulus-induced changes in Y_v_, particularly since basal Y_v_ partially accounts for inter-subject variation in the haemodynamic response to a stimulus. A range of MRI-based methods of measuring Y_v_ have been developed recently. Here, we use a method based on the change in phase in the MR image arising from the field perturbation caused by deoxygenated haemoglobin in veins. We build on the existing phase based approach (*Method I*), where Y_v_ is measured in a large vein (such as the superior sagittal sinus) based on the field shift inside the vein with assumptions as to the vein's shape and orientation. We demonstrate two novel modifications which address limitations of this method. The first modification (*Method II*), maps the actual form of the vein, rather than assume a given shape and orientation. The second modification (*Method III*) uses the intra and perivascular phase change in response to a known change in Y_v_ on hyperoxia to measure normoxic Y_v_ in smaller veins. *Method III* can be applied to veins whose shape, size and orientation are not accurately known, thus allowing more localised measures of venous oxygenation. Results demonstrate that the use of an overly fine spatial filter caused an overestimation in Y_v_ for *Method I*, whilst the measurement of Y_v_ using *Method II* was less sensitive to this bias, giving Y_v_ = 0.62 ± 0.03. *Method III* was applied to mapping of Y_v_ in local veins across the brain, yielding a distribution of values with a mode of Yv = 0.661 ± 0.008.

## Highlights

• We propose two modifications to MRI phase-based blood oxygenation measurements.
• A forward field calculation is used to relax the infinite cylinder approximation.
• Hyperoxia-based method overcomes vessel orientation and partial volume limitations.
• Local blood oxygenation measurements in small veins, showing regional heterogeneity

## Introduction

Venous cerebral blood oxygenation (Y_v_) is the haemoglobin oxygen saturation fraction in venous blood. The combination of in-vivo, non-invasive, MRI-based measurements of Y_v_ with other MR measures provides important haemodynamic information. For example, a measure of Y_v_ is required for hyperoxia-based calibrated blood oxygen-level dependent (BOLD) experiments as a measured (rather than assumed) value of basal oxygen extraction fraction (OEF) is preferred (Chiarelli et al., 2007, Driver et al., 2012), basal Y_v_ measurements can be combined with phase contrast MRI measures of cerebral blood flow (CBF) to calculate cerebral oxygen metabolism (Xu et al., 2009, Jain et al., 2010), and measures of Y_v_ have been used to explain inter-subject variability in fMRI results (Lu et al., 2008). The assessment of Y_v_ also has potential use in studying conditions where oxygen extraction is thought to be compromised, such as multiple sclerosis (Ge et al., 2012), traumatic brain injury (Shen et al., 2007) and carotid stenosis (Kavec et al., 2004).

MR-susceptometry-based measurements of Y_v_ estimate the susceptibility shift between a blood vessel and the surrounding tissue from phase maps, thus making such methods relatively simple and quick to implement. Whilst fully oxygenated blood has a similar magnetic susceptibility to the surrounding tissue, the susceptibility of deoxygenated haemoglobin (dHb) is sufficiently different to cause a field perturbation. This field perturbation results in a phase shift, the value of which depends on the amount of dHb and the orientation of the blood vessel. The magnetic susceptibility difference can be measured from this phase shift and hence Y_v_ can be estimated, assuming a value for haematocrit (Hct).

The most commonly used MR-susceptometry technique (Haacke et al., 1997, Fernandez-Seara et al., 2006, Jain et al., 2010, Fan et al., 2012) uses the intravascular phase shift arising from a long, straight vein, orientated approximately parallel to the direction of the static magnetic field (B_0_). If a vein is straight over a length much greater than its diameter, the field perturbation in and around it can be modelled as if it is an infinite cylinder; this approximation has been validated for MR-susceptometry in the superior sagittal sinus and jugular vein (Li et al., 2012). When the infinite cylinder approximation applies, the phase within the vein will be homogeneous, with an amplitude that depends on both the amount of dHb (and so Y_v_) and the vein's orientation to B_0_ (maximum where the vein is parallel to B_0_). The local extravascular phase variation will be maximum where the vein is perpendicular to B_0_ and zero where the vein is parallel to B_0_ (Boxerman et al., 1995).

The approach generally used to estimate Y_v_ is to select a long, straight vein, orientated approximately parallel to B_0_, such that the infinite cylinder approximation applies (Haacke et al., 1997, Fernandez-Seara et al., 2006, Jain et al., 2010). The chosen vein also needs to be sufficiently large such that an intravascular region free from partial volume effects can be selected to avoid diluting the estimated Y_v_. Therefore this approach yields a global measure of Y_v_, limited to a small number of large veins, each draining large portions of the cortex. Recent attempts to select smaller, more local veins (Fan et al., 2012) require manual selection of voxels which are entirely intravascular, which is potentially subject to observer error or bias. An alternative approach using susceptibility mapping was recently proposed, which exploits intravascular and extravascular phase data to estimate the tissue–vein susceptibility difference and measure Y_v_ in smaller veins (Haacke et al., 2010). However, this approach also potentially has the same partial voluming effects.

Alternatively, Y_v_ can be measured by exploiting the effect of dHb on the transverse relaxation rate of blood, T_2,blood_. Measurements of T_2,blood_ in the superior sagittal sinus have been used to estimate global Y_v_ (Oja et al., 1999, Lu and Ge, 2008, Jain et al., 2013), whilst Y_v_ has been mapped using T_2_ measurements in which velocity encoding has been applied to select the venous blood signal and suppress static tissue (Bolar et al., 2011, Guo and Wong, 2012). These approaches require prior calibration to estimate Y_v_ from T_2,blood_, based on in-vitro T_2_ measurements made on blood samples over a range of Y_v_ and Hct values. Gradient-echo sampling of spin-echo (GESSE) has also been used to map Y_v_ (He and Yablonskiy, 2007), where the shape of the sampled transverse relaxation curve is dependent on both oxygen extraction fraction (OEF) and cerebral blood volume (CBV), though this requires fitting of the data to a complex model to disentangle OEF from CBV.

More recently, an alternative method has been proposed which builds on calibrated BOLD methodology (Bulte et al., 2012, Gauthier and Hoge, 2012, Wise et al., 2013). Hypercapnia (Davis et al., 1998) and hyperoxia (Chiarelli et al., 2007) calibration models are combined to map OEF (and hence Y_v_) based on measurements of BOLD and CBF in response to hypercapnia and hyperoxia. This approach is time consuming since these measurements are needed at hypercapnia, hyperoxia and normocapnic normoxia.

In this study we compare three methods of calculating Y_v_ using MR phase measures. *Method I*: the existing approach which assumes the infinite cylinder approximation for a vein that is approximately parallel to B_0_, this provides an estimate of Y_v_ by considering the field shift inside a large vein, here taken to be the superior sagittal sinus. *Method II*: overcomes any assumption about the shape and orientation of the vein by using a forward field calculation (Marques and Bowtell, 2005) of the intravascular field perturbation based on the actual shape of the vein. *Method III*: the change in intra and perivascular phase distribution to a known change in Y_v_ on hyperoxia is used to calculate Y_v_ on normoxia. Since this approach does not require a model of the expected intra or extravascular phase distribution (from the forward model or infinite cylinder model), it is suitable for veins of any shape and orientation, and can be applied to smaller veins as it is not biased by partial voluming. Therefore, this method could be applied to measure Y_v_ in local veins, as apposed to providing just a global measurement.

## Methods

Eight healthy volunteers (7 male, 1 female; age range 22–32 years) participated in this study. Ethical approval was given by the University of Nottingham Medical School Ethics Committee and all subjects gave informed written consent prior to participating. Scanning was performed on a Philips Achieva 7 T system, with a head volume transmit coil and 32 channel head receive coil. Whole-head, flow-compensated, 3D FLASH data (TE/TR = 5/9.5 ms, SENSE = 2.25 (AP)) were acquired at 0.65 mm isotropic resolution (208 × 208 × 100 mm FOV) in 3.5 min. In six subjects, 3D FLASH data was collected at both normoxia and hyperoxia. Details of the respiratory paradigm are provided in Section Respiratory paradigm. In a further two subjects, the reproducibility of the estimate of Y_v_ from intravascular measurements was assessed by repeating the 3D FLASH acquisition multiple times (6 repeats for the first subject, 7 repeats for the second subject, each repeat separated by a 2 min pause), with the subject breathing room air. The numbers of repeats were determined by the total time that the subject felt that they could comfortably remain still. In addition, for all subjects a multi-echo 3D FLASH dataset was acquired at normoxia, comprising 3 echoes at TE = 5/10/15 ms, TR = 21 ms, SENSE = 2.25 (AP)/1.4 (FH), with the same spatial resolution and coverage to allow identification of veins.

### Respiratory paradigm

A feed-forward, low gas flow system (RespirAct™, Thornhill Research Inc., Toronto, Canada) and a sequential gas delivery (SGD) breathing circuit (Banzett et al., 2000, Slessarev et al., 2007) were used to target end-tidal PCO_2_ (P_ET_CO_2_) and PO_2_ (P_ET_O_2_) independently (Slessarev et al., 2007). Source gases used by the system were O_2_, air, and two gas blends of N_2_, CO_2_ and O_2_, so that all source gases contained safe concentrations of O_2_. The RespirAct™ uses the approach of Slessarev et al. (2007) to calculate the required flows of these source gases into the SGD breathing circuit to attain the targeted P_ET_CO_2_ and P_ET_O_2_ values. For the normoxia condition, both P_ET_O_2_ and P_ET_CO_2_ were maintained at the subject's resting values (~ 110 mm Hg and ~ 40 mm Hg, respectively). For the hyperoxia condition, P_ET_O_2_ was targeted at 500 mm Hg, whilst P_ET_CO_2_ was maintained at the resting value.

### Analysis

**Fig. 1:**
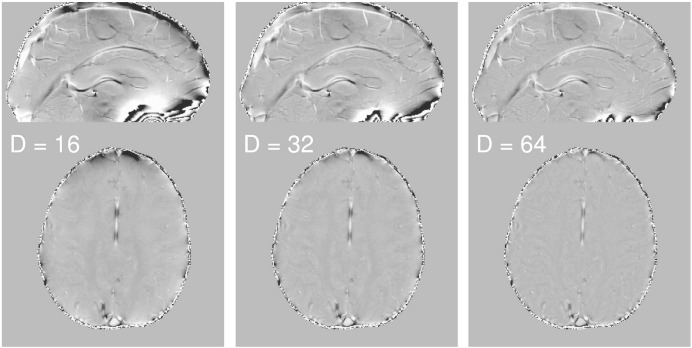
Example normoxia phase data after spatial filtering with a complex Hanning filter diameter of D = 16, D = 32 and D = 64 pixels in k-space.

A complex 2D Hanning spatial filter was used to high-pass filter the phase data (Wang et al., 2000). Whilst both Hanning filtering (Fernandez-Seara et al., 2006, Fan et al., 2012) and polynomial fitting (Jain et al., 2010) techniques have been used previously to remove large scale phase inhomogeneities at lower field strengths, the polynomial fitting approach lacked sufficient flexibility to accurately account for the greater inhomogeneities found at 7 T. Three filter sizes were compared, with the Hanning filter kernel diameters of D = 16, 32 and 64 pixels in k-space, respectively. Fig. 1 shows the results of applying each of these filter sizes to phase data collected at normoxia. The spatial filter removes large-scale phase changes due to external sources and is particularly relevant for data collected at hyperoxia, where increased oxygen in the oral cavity and frontal sinus leads to an increased field inhomogeneity across the brain (Pilkinton et al., 2011). Therefore, the finest filter (D = 64) was applied to both hyperoxia and normoxia phase data for *Method III*. For each single echo 3D FLASH dataset, the magnitude data were registered to the first echo magnitude image of the multi-echo 3D FLASH dataset, using FLIRT (FSL, fMRIB, Oxford, UK) and the resulting transformations applied to the corresponding filtered phase data.

#### Measurement of Y_v_ using intravascular phase (*Methods I* and *II*)

Y_v_ was calculated based on the phase shift (Δϕ_IV_) between the surrounding tissue and the vein, which was converted to a field shift (ΔB) using

(1) $$\mathrm{\Delta B}=\frac{\Delta \phi_{\mathrm{IV}}}{\gamma \cdot \mathrm{TE}},$$

where γ is the proton gyromagnetic ratio and TE is the echo time. The susceptibility shift (Δχ_IV_) of the vein relative to the surrounding tissue was calculated from ΔB, using

(2) $$\mathrm{\Delta B}=A\cdot B_{0}\cdot {\Delta \chi }_{\mathrm{IV}},$$

where B_0_ = 7 T and *A* is a scaling factor which depends on the vein's geometry which was calculated in two ways, as described in Sections Method I: infinite cylinder approximation and Method II: forward field calculation, Sections Method I: infinite cylinder approximation and Method II: forward field calculation. Δχ_IV_ is related to Y_v_ by

(3) $${\Delta \chi }_{\mathrm{IV}}=\mathrm{Hct}\cdot \left(1-Y_{V}\right)\cdot {\Delta \chi }_{\mathrm{do}},$$

where Hct is haematocrit (Hct = 0.4 assumed here), and Δχ_do_ = 3.32 × 10^− 6^ (SI units, 0.264×10^− 6^ in cgs units) is the volume susceptibility difference between fully deoxygenated and fully oxygenated haemoglobin (Spees et al., 2001, Jain et al., 2012). Eq. (3) is based on the assumption that tissue has a similar susceptibility to fully oxygenated haemoglobin, as has been assumed in previous MR-susceptometry studies of blood oxygenation (Haacke et al., 1997, Fernandez-Seara et al., 2006, Jain et al., 2010, Fan et al., 2012). Thus Y_v_ can be estimated from

(4) $$Y_{V}=1-\frac{\Delta \phi_{\mathrm{IV}}}{A\cdot \gamma \cdot \mathrm{TE}\cdot B_{0}\cdot \mathrm{Hct}\cdot {\Delta \chi }_{\mathrm{do}}}.$$

**Fig. 2:**
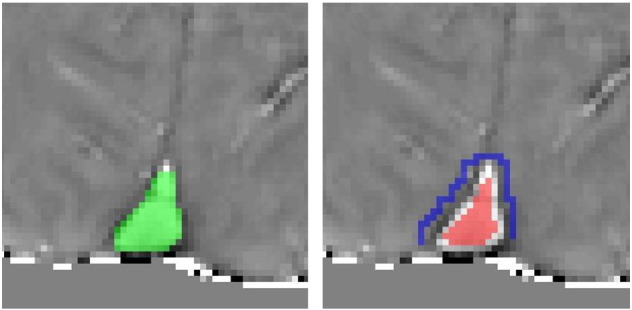
Example intravascular (red) and reference (blue) ROI, based on the manually drawn sagittal sinus ROI (green).

To calculate Δϕ_IV_ in *Methods I* and *II*, the difference in filtered phase was found between an intravascular region of interest (ROI) within the superior sagittal sinus and a local reference ROI in tissue, as illustrated in Fig. 2. A sagittal sinus ROI was drawn manually around the sinus on the first echo magnitude image of the multi-echo dataset, extending from the confluence of sinuses to the point at which the sinus curves to approximately 30° from the B_0_ direction. This sagittal sinus ROI was also required for the forward field calculation described in Section Method II: forward field calculation. A 30° limit was chosen firstly to provide a repeatable boundary for the ROI, and secondly since the infinite cylinder approach has been shown to be a reasonable approximation up to tilt angles of 30° (Li et al., 2012). Five slices were selected, where the sagittal sinus was approximately parallel to B_0_. An intravascular ROI was formed by eroding the sagittal sinus ROI by one voxel (3 × 3 kernel) over these 5 slices. A reference ROI (a ring of tissue surrounding the sagittal sinus) was formed by first dilating the sagittal sinus ROI by two voxels (5 × 5 kernel) and then subtracting the sagittal sinus ROI dilated by one voxel (3 × 3 kernel). To avoid edge artefacts caused by the spatial filter, these ROIs were constrained by applying a whole brain mask (formed using BET (FSL)) eroded by 3 voxels (7 × 7 kernel). Δϕ_IV_ was calculated from the difference in the average filtered phase data between the intravascular and reference ROIs.

##### *Method I*: infinite cylinder approximation

The infinite cylinder approximation (*Method I*) assumes that the vein is straight over the region of interest and that the length of the vein is much greater than its diameter. In this case, the scaling factor *A* in Eq. (4) is given by

(5) $$A=\frac{\left(3\cos^{2}\theta -1\right)}{6},$$

where θ is the angle of the cylinder's long axis relative to the direction of B_0_. A section of the sagittal sinus which was parallel to B_0_ (θ = 0) was chosen, such that *A* = 1/3, providing maximal Δϕ_IV_. The extravascular phase distribution is close to zero where the sagittal sinus is parallel to B_0_, minimising any contribution to the local reference ROI.

##### *Method II*: forward field calculation

**Fig. 3:**
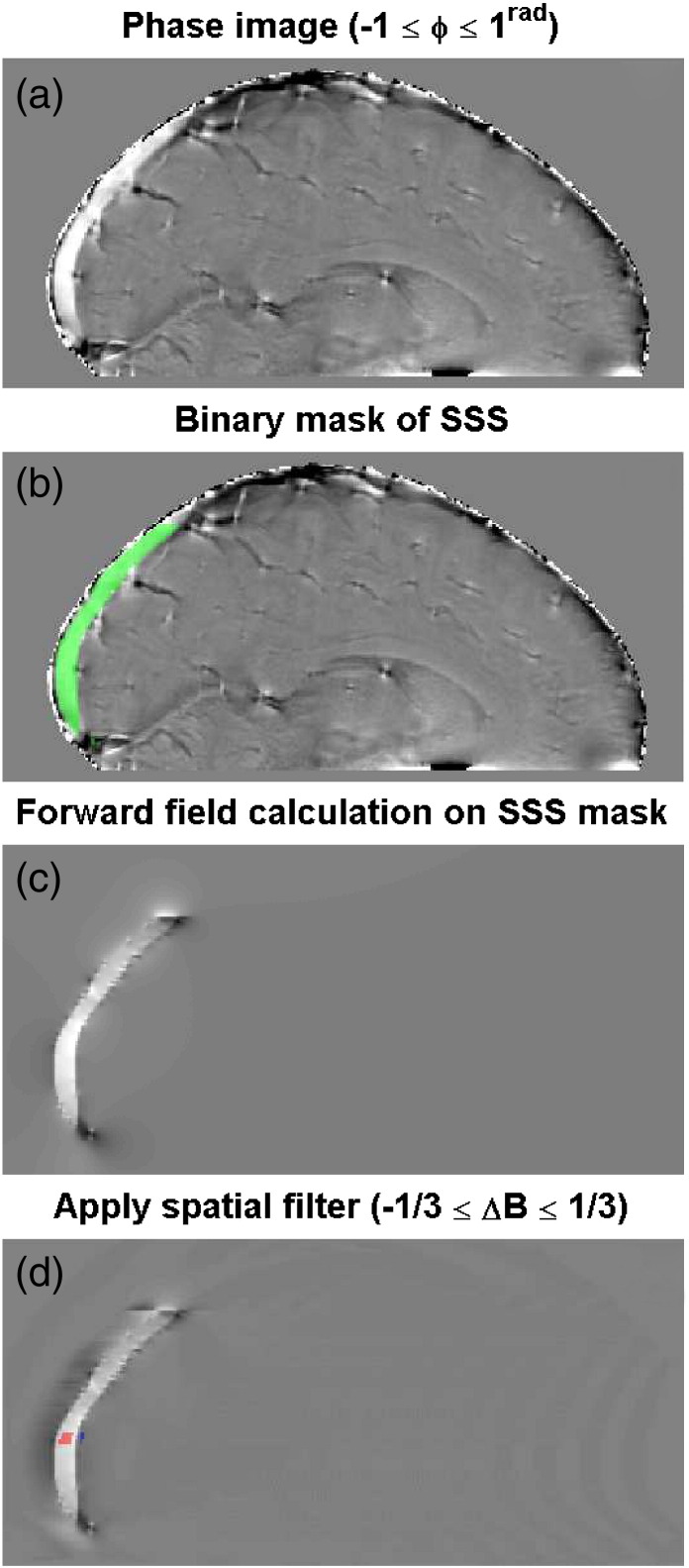
The analysis steps for the forward field calculation. A manually drawn superior sagittal sinus (SSS) binary mask (b, green ROI) is used to form a field perturbation map (c), which is then spatial filtered (d) using the same filter as applied to the phase data (a). The intravascular (red) and reference (blue) ROIs in (d) are the same as in Fig. 2.

The second approach (*Method II*) uses a forward field calculation (Marques and Bowtell, 2005) which takes account of the exact shape of the superior sagittal sinus (SSS), as illustrated in Fig. 3. An ROI was drawn around the superior sagittal sinus, from the confluence of sinuses to where the sinus curves to approximately 30° away from the B_0_ direction, based on the first-echo magnitude image of the multi-echo dataset. This was then used to form a susceptibility map, with unit susceptibility difference between the ROI and elsewhere. The forward field calculation employs a discrete Fourier transform, so a large matrix size was used to ensure that aliasing artefacts were minimised and located at the edge of the padded matrix, away from the area of interest around the sagittal sinus ROI (Marques and Bowtell, 2005). The ROI may be located off centre in the original image, so the original matrix was included in the padded matrix to prevent discontinuities when unpadding (i.e. returning to the original image space), which would affect spatial filtering (detailed below). Therefore the matrix containing the sagittal sinus ROI mask was zero-padded to an N × N × N matrix, centred on the ROI, where N was at least twice the length of the ROI, and included the entire original image matrix.

The forward field calculation in k-space was used to estimate the field in the vessel. A 3D discrete fast Fourier transform (MATLAB, The MathWorks, Natick, USA) was performed on the padded matrix, the field perturbation calculated based on Eq. (6) in Marques and Bowtell (2005), and then an inverse 3D discrete fast Fourier transform was performed. The orientation of the matrix with respect to B_0_ is required in the calculation, which was found by combining the angles recorded when planning the scan geometry with the transformations applied to register the image to the multi-echo data.

The resulting field perturbation map was unpadded, so as to be aligned with the original image. The spatial filter applied to the phase data (see above) was also applied to this field perturbation map. The filtered map was then used to calculate the scaling factor *A*, as the difference in field perturbation between the sagittal sinus and the surrounding tissue based on Δχ = 1, which is then used in Eq. (4) to calculate Δχ.

#### *Method III*: measurement of Y_v_ using hyperoxia intra and perivascular phase contrast

Since haemoglobin in arterial blood is close to having full oxygen saturation at normoxia, the extra arterial oxygen that is present on hyperoxia is dissolved in blood plasma. When blood enters the capillaries, dissolved oxygen is taken up into cerebral tissue as readily as that bound to haemoglobin, so Y_v_ is increased. The change in Y_v_ on hyperoxia was calculated using an existing model, see below. Therefore a controlled level of hyperoxia was used to generate a controlled change in Y_v_, to perturb the amplitude of the phase distribution generated by venous dHb.

This method assumes that the phase distribution in and around a vessel at normoxia is dominated by the effect of intravascular dHb and thus provides a template for the phase change on hyperoxia due to a change in Y_v_. This is an extension and simplification of the method that we recently introduced to measure Y_v_ at normoxia (Driver et al., 2012). Thus, the hyperoxia phase distribution (*ϕ_HO_*(*r*)) was fitted to the normoxia phase distribution (*ϕ_NO_*(*r*)), according to

(6) $$\phi_{\mathrm{HO}}\left(r\right)=a\cdot \phi_{\mathrm{NO}}\left(r\right)+k,$$

where *a* is the fitting parameter and the subscripts *HO* and *NO* refer to hyperoxia and normoxia, respectively. A conventional linear least-squares minimisation approach was not appropriate in this case, since both coordinates (Δϕ*_HO_* and Δϕ*_NO_*) have associated errors. *a* was therefore found by using York's solution to a linear least-squares fit with errors in both coordinates (Reed, 1989, Reed, 1992). This linear fitting approach can fit the gradient (*a*) independently of the intercept (*k*), so any non-local phase offsets between *ϕ_HO_* and *ϕ_NO_* will not introduce bias (in the limit where the offset is constant across the ROI). Thus, assuming that the phase distribution in the ROI is dominated by the susceptibility of the vein,

(7) $$a=\frac{{\Delta \chi }_{\mathrm{HO}}}{{\Delta \chi }_{\mathrm{NO}}},$$

where Δχ is the susceptibility difference between the vein and the surrounding tissue. This equation makes no assumptions about the spatial nature of the field shift around the vein. Assuming that tissue has the same susceptibility as fully oxygenated haemoglobin, Δχ can be related to Y_v_ by

(8) $${\Delta \chi }_{\mathrm{NO}}=\mathrm{Hct}\cdot \left(1-Y_{V}\right)\cdot {\Delta \chi }_{\mathrm{do}},$$

(9) $${\Delta \chi }_{\mathrm{HO}}=\mathrm{Hct}\cdot \left(1-Y_{V}-{\mathrm{\Delta Y}}_{h}\right)\cdot {\Delta \chi }_{\mathrm{do}},$$

where ΔY_h_ is the change in venous blood oxygenation due to hyperoxia. Eqs. (8), (9) can be substituted into Eq. (7) and rearranged to give

(10) $$Y_{V}=1-\frac{{\mathrm{\Delta Y}}_{h}}{\left(1-a\right)}.$$

*ϕ*(*r*) was assessed over an ROI selected as follows. The multi-echo magnitude data were combined into an R_2_* map using a voxel-wise linear regression (MATLAB). Veins were identified by applying a threshold of R_2_* > 100 s^− 1^. Additionally, the whole-brain mask, eroded by 5 voxels (11 × 11 kernel), was applied to exclude voxels close to the edge of the brain due to edge artefacts from the spatial filter. Individual veins were indexed by clustering the binary thresholded map (convolution with a 3 × 3 × 3 kernel). This segmentation method often included the inter-hemispheric fissure, so this was excluded by removing any clusters which included 1200 or more voxels. An ROI was defined for each vein by dilating the binary map by one voxel (using a 3 × 3 × 3 kernel). This approach was found to be optimal for small veins, where it covered the majority of the vein's phase distribution, without including a significant number of voxels where Δϕ dropped into the noise.

A model proposed by Chiarelli et al. (2007) was used to convert P_ET_O_2_ measurements into ΔY_h_, assuming that arterial PO_2_ (PaO_2_) can be approximated by P_ET_O_2_ (i.e. PaO_2_ = P_ET_O_2_). Whilst this assumption is not generally true, it is reasonable for the gas delivery method used here (see *Estimating q_h_ section of* Driver et al. (2012)). Assuming that the amount of oxygen extracted from the capillaries is constant between normoxia and hyperoxia, then

(11) $${\mathrm{\Delta Y}}_{h}=\frac{\psi \cdot \left[\mathrm{Hb}\right]\cdot \mathrm{\Delta Sa}O_{2}+\varepsilon \cdot \Delta \mathrm{Pa}O_{2}}{\psi \cdot \left[\mathrm{Hb}\right]},$$

adapted from Eq. (11) in Driver et al. (2012), where *ψ* = 1.34 ml (O_2_)/g is the oxygen carrying capacity of haemoglobin, [*Hb*] = 15 g/dl_blood_ is the concentration of haemoglobin and *ε* = 0.0031 ml/dl_blood_/mm Hg is the solubility coefficient of oxygen in blood (values obtained from Chiarelli et al. (2007)). *ΔPaO*_2_ is the difference in PaO_2_ between hyperoxia and normoxia, whilst *ΔSaO*_2_ is the difference in arterial haemoglobin oxygen saturation (SaO_2_) between hyperoxia and normoxia. SaO_2_ was calculated at hyperoxia and normoxia from PaO_2_, based on the oxygen dissociation in blood, described by a widely accepted model (Severinghaus, 1979):

(12) $${\mathrm{SaO}}_{2}={\left(\frac{23400}{{\mathrm{PaO}}_{2}^{3}+150{\mathrm{PaO}}_{2}}-1\right)}^{-1}.$$

## Results

### Intravascular measurement of Y_v_ (*Methods I* and *II*)

**Fig. 4:**
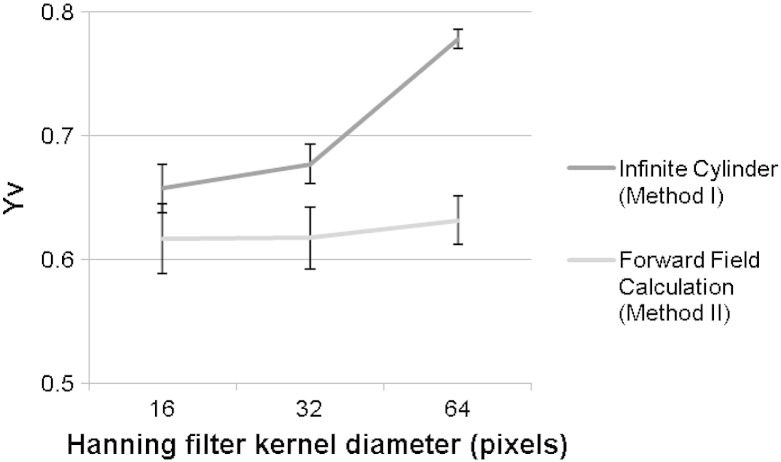
Mean Y_v_ across subjects (SEM error bars) based on intravascular phase after spatial filtering with each of the three different complex Hanning filter widths. Results based on the infinite cylinder approximation (*Method I*) are shown in black and the forward field calculation (*Method II*) is shown in grey.

Measurements of Y_v_ in the superior sagittal sinus for each of the three Hanning filter diameters (D = 16, 32 and 64) are shown in Fig. 4, for both the infinite cylinder (*Method I*) and forward field calculation (*Method II*) approaches. Whilst the use of the finer spatial filter (D = 64) in *Method I* leads to an overestimation of Y_v_ (due to the filter being of a similar length scale to the size of the superior sagittal sinus), this overestimation was much smaller for *Method II*. The value of Y_v_ calculated using the forward field calculation was less than that found from the infinite cylinder approximation, due to the value of *A* being less than 1/3 in every instance (each filter size for each subject). For example, *A* = 0.303 ± 0.006 (mean ± SEM, 8 subjects) for the D = 16 filter. Individual subject Y_v_ values are shown in Table 1 for the D = 16 filter, with mean Y_v_ values of 0.66 ± 0.05 for *Method I* and 0.62 ± 0.07 for *Method II* (mean ± SD, 8 subjects).

**Table 1:** Individual subject Y_v_ values in the sagittal sinus for *Method I* (*infinite cylinder approximation*) and *Method II* (*forward field calculation*), results are shown for the D = 16 filter.

Individual subject Y_v_ values		
*Method I* (sagittal sinus)	*Method II* (sagittal sinus)	*Method III*^a^ (averaged over small veins)
0.712	0.705	0.652
0.620	0.582	0.666
0.682	0.644	0.647
0.678	0.654	0.687
0.651	0.622	0.638
0.581	0.490	0.676
0.607 ± 0.015^b^	0.545 ± 0.017^b^	
0.728 ± 0.020^b^	0.697 ± 0.023^b^	
0.66 ± 0.05^c^	0.62 ± 0.07^c^	0.66 ± 0.02^c^

a Mode across veins shown for *Method III* (*using hyperoxia phase contrast*).

b The two reproducibility subjects are marked with (mean ± SD across repeats).

c Mean ± SD Y_v_ across subjects.

The coefficient of variation (CoV) in the estimate of Y_v_ was calculated for the reproducibility data. For the forward field calculation with the D = 16 filter, a CoV of 3.0% and 3.4% was found for each of the two subjects. Similar CoVs were found for the D = 32 and D = 64 filters and between the forward field calculation and infinite cylinder approaches (CoV range 1.5–3.6%).

### Measurement of Yv using hyperoxia phase contrast (*Method III*)

P_ET_O_2_ increased by 320 ± 20 mm Hg (mean ± SEM, 6 subjects), leading to a calculated increase in venous blood oxygenation of ΔY_h_ = 0.066 ± 0.003. It is worth noting that the hyperoxia was not accompanied by significant hypocapnia; average P_ET_CO_2_ was 38.9 ± 1.0 mm Hg at normoxia and 38.6 ± 1.3 mm Hg at hyperoxia (both mean ± SEM across subjects), with the change between normoxia and hyperoxia ranging from + 1.21 to − 1.69 mm Hg (increase in 2 subjects/decrease in 4 subjects).

**Fig. 5:**
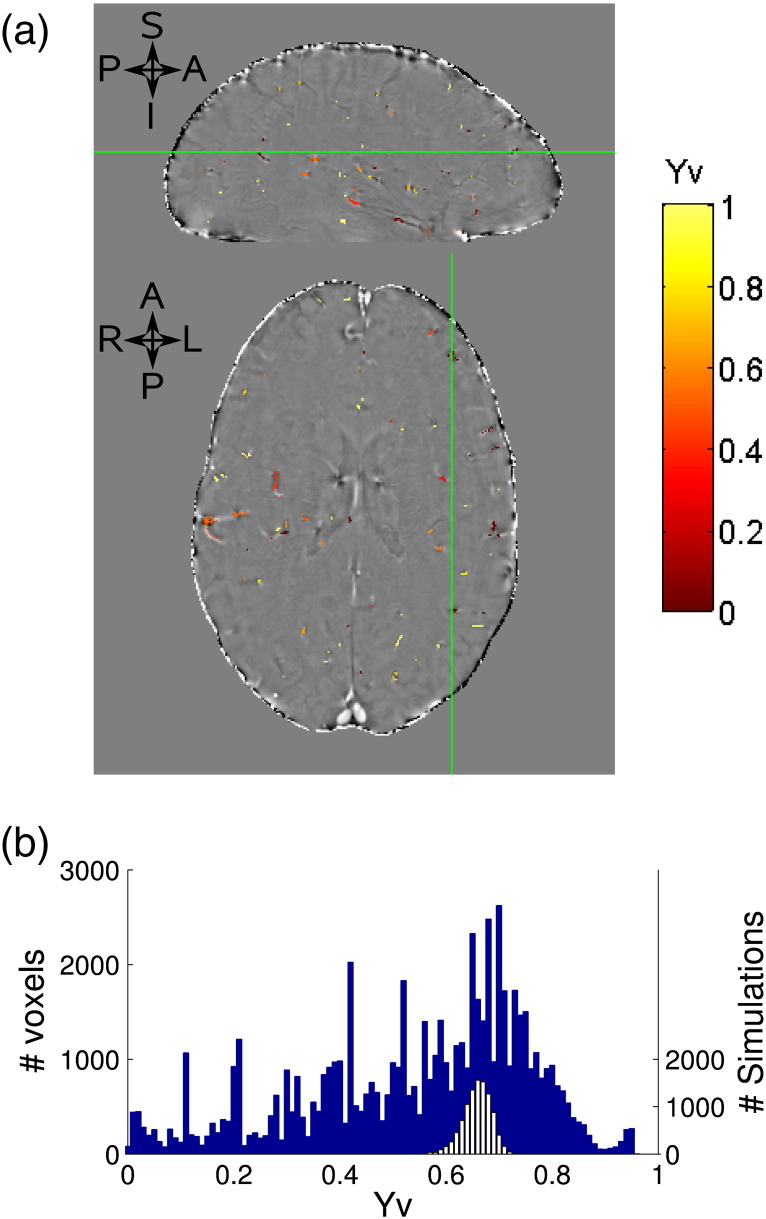
Results for the hyperoxia phase contrast method (*Method III*). (a) Single subject map of Y_v_, overlaid on the filtered (D = 64) normoxia phase data. Sagittal and axial slices are shown, whilst the green line shows the location of the slice position of the other orientation. (b) Histogram showing Y_v_ values for each vein across all subjects, where each vein is weighted by the number of voxels in that vein (blue histogram, left axis). Monte Carlo simulation results of this method are also shown (white histogram, right axis).

Fig. 5a shows an example map of Y_v_ calculated for each detectable vein, overlaid on the normoxia phase image. Note that due to the simple clustering algorithm, a small number of veins were selected in a cluster in the inter-hemispheric fissure. Therefore Y_v_ was not calculated for these veins. The distribution of Y_v_ values across all veins in all 6 subjects is shown in a histogram in Fig. 5b, since the data is not normally distributed, the estimate of Y_v_ in each subject was characterized by the mode of the distribution. Individual subject Y_v_ modes are shown in Table 1, with the mean of these individual subject values being Y_v_ = 0.66 ± 0.02 (mean ± SD, 6 subjects).

Monte Carlo simulations were performed to assess the effect of noise propagation from the fit for *a* into the Y_v_ calculation (Eq. (10)). 10,000 pairs of normoxia and hyperoxia phase distributions with the same order of SNR (= 20) as the actual data in an ROI of 200 voxels were simulated. An example of these simulations for a value of Y_v_ = 0.66 is shown in Fig. 5b (white histogram), demonstrating good precision in the calculated value of Y_v_, but a slight skew towards smaller Y_v_ values due to the noise propagation through the reciprocal relationship described in Eq. (10). This skew was found to increase at lower Y_v_ values and is also seen in the real data (blue histogram). The real data has a larger variance than the simulated data, partly due to variation in the sizes of ROIs used in the real fits, and also due to expected heterogeneity in Y_v_ between veins and between subjects.

There was no significant difference in the results from the three methods (p = 0.3 single effect ANOVA) when the D = 16 filter was used for *Methods I* and *II*.

## Discussion

In this study we compare three approaches for measuring venous blood oxygenation based on mapping the phase distribution caused by deoxyhaemoglobin in the vein. The intravascular phase in the superior sagittal sinus was used to calculate Y_v_, either using an infinite cylinder approximation (*Method I*) or a forward field calculation using the actual shape of the individual superior sagittal sinus (*Method II*). In addition, a new approach for mapping Y_v_ based on measuring the change in the phase distribution around small veins on hyperoxia is demonstrated (*Method III*). There was no significant difference in Y_v_ measured from the three methods (considering the coarsest spatial filter was used for *Methods I* and *II*), as assessed by a 1-way ANOVA, although it should be noted that *Methods I* and *II* had larger inter-subject variance in Y_v_ compared to *Method III*.

However there was a marked trend for *Method I* to measure larger values of Y_v_ than *Method II*. This is expected since the forward field calculation (*Method II*) captures individual variations in the shape and orientation of each subject's sagittal sinus, unlike the infinite cylinder approach. As a result, in the forward field calculation the scaling factor *A* was consistently found to be less than 1/3 (the value assumed for the infinite cylinder approximation: *Method I*), leading to smaller estimated values of Y_v_. On the other hand there is a human error associated with manually drawing around the sagittal sinus and hence the forward field calculation method is more labour intensive than the infinite cylinder approach. Spatial filtering may also be expected to affect the two methods differently. The choice of spatial filter is particularly important when considering large veins, since finer spatial filters will reduce the vein–tissue phase difference when the filter length scale is similar to the size of the vein, causing an overestimation in Y_v_ (Langham et al., 2009). However the effect of the filtering is somewhat overcome in *Method II* since the forward field calculation maps are also filtered. This was confirmed by the relative stability of Y_v_ measurements across filter size for *Method II* compared to *Method I*. Therefore on balance it is expected that *Method II* will be more accurate than *Method I*, although cross-validation with an independent technique is required to confirm this.

The method for selecting a reference ROI for *Methods I* and *II* was chosen to be reproducible across subjects and to ensure that the reference ROI was local to the intravascular ROI. On visual inspection this reference ROI did not appear to overlap with the extravascular field surrounding the sagittal sinus. A distance of approximately 5 mm between intravascular and reference regions has been used previously compared to 1.95 mm used here (Li et al., 2012). In future work it would be useful to investigate the effect of varying the position of the reference ROI. For *Method I*, as well as being orientated approximately parallel to the static magnetic field, the portion of the sagittal sinus to be investigated should also be fairly straight, to maximise the accuracy of the infinite cylinder approximation.

In this work we have assumed that tissue has a similar susceptibility to fully oxygenated haemoglobin, an approach taken previously. Studies measuring the susceptibility difference Δχ_do_ suggest that fully oxygenated haemoglobin is slightly diamagnetic relative to water (Weisskoff and Kiihne, 1992, Spees et al., 2001, Jain et al., 2012). Tissue susceptibility is difficult to measure and is likely to vary across brain regions and subjects. The effect of removing the assumption of tissue susceptibility being equal to that of fully oxygenated haemoglobin will introduce an extra term (+ Hct · Yv · Δχ_oxy_) into Eq. (3) and equivalent terms in Eqs. (8), (9), where Δχ_oxy_ is the susceptibility difference between fully oxygenated haemoglobin and tissue. These lead to an additional term + Δχ_oxy_ / Δχ_do_ in Eqs. (4), (10). For example, using the value of Δχ_oxy_ = − 0.21 × 10^− 6^ (SI units, or − 0.017×10^− 6^ in cgs units) based on the susceptibility difference between water and fully oxygenated haemoglobin (Spees et al., 2001), the value for Y_v_ would be 0.06 smaller if tissue susceptibility were equal to that of water, as opposed to that of fully oxygenated haemoglobin. This is expected to be an upper limit on any systematic error in Y_v_, since tissue is also likely to be slightly diamagnetic relative to water due to the presence of myelin (Liu et al., 2011, Lee et al., 2012, Wharton and Bowtell, 2012).

The use of an assumed value for Hct could lead to a systematic error in Y_v_, especially when studying pathologies where Hct may be perturbed. This limitation is not restricted to susceptometry-based methods, but is also true for the other MRI-based oximetry techniques mentioned in the Introduction section. This limitation can be overcome through direct measurement of Hct from blood samples, although some variations through the circulatory system may remain (Yang et al., 2001, Baskurt et al., 2006).

Whilst the intravascular methods (*Methods I* and *II*) can only be used on large veins, whose size, shape and orientation can be clearly determined, the hyperoxia-based method (*Method III*) uses the normoxia phase distribution as a model of the pattern of phase change expected on hyperoxia. Since the phase distribution of a vein extends into the surrounding tissue, Y_v_ can be mapped from veins whose diameters are smaller than the voxel size, and voxels that are partially intravascular and partially extravascular do not have to be discarded, as they do for the intravascular methods (*Methods I* and *II*). Whilst a simple, automated approach was used here for vein identification and for fitting the phase distribution in hyperoxia to the normoxia phase distribution, in the future, more sophisticated techniques could improve specificity. For example, in this work the broad range of veins selected meant that the ROI selection was not optimal for every vein investigated, whereas a more targeted study of specific veins, with the ROI selection individually optimised to the specific extent of the vein's field distribution, would achieve an improved SNR in Y_v_.

The results from *Method III* cannot be directly compared with *Methods I* and *II* since the hyperoxia contrast method was not applied in the superior sagittal sinus due to the use of extravascular signal and the proximity of the sagittal sinus to edge artefacts arising from spatial filtering. Whilst small residual contributions from edge artefacts have little effect on the absolute IV phase measurements in *Methods I* and *II*, *Method III* considers small changes in the phase distribution, which are more sensitive to bias induced by these edge artefacts which may differ between normoxia and hyperoxia phase images. Further work is required to overcome this limitation. Nonetheless there was a trend for *Method III* to measure higher oxygenation than *Method II*, as might be expected in these smaller veins.

Care must be taken with image registration when mapping the hyperoxia phase distribution onto the normoxia phase distribution. Significant rotations will change the vein orientation with respect to B_0_, changing the phase distribution. For this study, the maximum rotation between normoxia and hyperoxia scans was less than 4° with respect to B_0_ and less than 1° in the plane perpendicular to B_0_, so it was considered to have an insignificant effect on the phase distribution.

The hyperoxia-based method assumes that cerebral blood volume (CBV), cerebral blood flow (CBF) and cerebral metabolic rate of oxygen consumption (CMRO_2_) remain constant between the normoxia and hyperoxia scans. This is important since changes in these parameters will affect ΔY_h_, introducing a bias into Y_v_ (propagating linearly, according to Eq. (10)). CBV and CBF can be maintained during hyperoxia by employing isocapnic hyperoxia (Croal et al., 2012) to prevent systematic changes in arterial CO_2_ concentrations (CO_2_ is a potent vasodilatory stimulus). An isocapnic gas challenge can either be presented using the prospective method that we use here (Slessarev et al., 2007), or by dynamic end-tidal forcing (Wise et al., 2007). Whilst previous calibrated BOLD studies have assumed constant CBV, CBF and CMRO_2_ on isocapnic hyperoxia (Mark et al., 2011, Driver et al., 2012), recent work (Xu et al., 2012) measured a decrease in CMRO_2_ with hyperoxia. However, it should be noted that their study preceded a hyperoxia condition with a hypoxia condition, and this may have affected the results since it is known that cerebral lactate concentrations remain elevated for more than 10 min after the end of a hypoxia challenge (Harris et al., 2013).

The hyperoxia-based method for measuring Y_v_ uses a model to estimate a value of ΔY_h_ from measured P_ET_O_2_, as we proposed previously (Driver et al., 2012). This model is adapted from that used for hyperoxia-based calibrated BOLD experiments (Chiarelli et al., 2007). By calculating absolute, rather than relative changes in Y_v_, the model is independent of OEF. A single value of ΔY_h_ is assumed across the whole brain for each subject, which is reasonable since arterial blood oxygenation should be homogeneous across brain regions. The method is insensitive to local variations in oxygen extraction, so long as oxygen extraction does not change between normoxia and hyperoxia.

Another assumption used in *Method III* is that tissue susceptibility does not change due to hyperoxia, whereas in practice any changes in tissue PO_2_ will change the tissue susceptibility and the vein–tissue susceptibility difference. Tissue PO_2_ can be estimated based on measured P_ET_O_2_ and an assumed normoxia OEF value (Schwarzbauer and Deichmann, 2012). The change in tissue PO_2_ (ΔP_t_O_2_) decreases with increasing normoxic OEF. Based on a P_ET_O_2_ change from 110 to 500 mm Hg (approximate targets for this study), ΔP_t_O_2_ = 61.5 mm Hg will occur when a low OEF of 0.3 is assumed. This would cause an increase in tissue susceptibility Δχ = 3.3 × 10^− 9^ (SI units), which is more than 30 times less than the vein susceptibility Δχ due to ΔY_h_. Smaller tissue Δχ values will occur for higher normoxic OEF values (e.g. Δχ = 2.6 × 10^− 9^ for an OEF of 0.4), so this will have an insignificant effect on the value of Y_v_ measured.

In conclusion, the intravascular phase approach based on an infinite cylinder approximation is the simplest to implement and can be performed on a single 2D slice (minimising acquisition times), however it is the most susceptible to the effects of the spatial filter removing phase contrast. Y_v_ measurements made using the intravascular phase approach based on a forward field calculation are less sensitive to the spatial filter, however this approach is more user intensive, as the shape of the vein must be mapped out. Both of these intravascular phase approaches require the vein of interest to be sufficiently large that intravascular voxels that are free from partial voluming can be selected. These methods also require assumptions to be made as to the shape of the phase distribution, involving knowledge of the vein's size, shape and orientation. The hyperoxia phase contrast-based approach provides a measurement of Y_v_ that does not require any prior knowledge of the vein's geometry, instead it compares maps of the hyperoxia phase distribution with those of the normoxia phase distribution. Whilst this method requires the delivery of isocapnic hyperoxia, it shows promise in allowing the measurement of Y_v_ in smaller veins, which would be susceptible to partial volume issues when using other phase-based approaches, thus allowing the study of regional variations in OEF.
